# Cellular expression domains of type 3 deiodinase in the meninges, choroid plexus, tanycytes and barrier tissues of the brain

**DOI:** 10.3389/fendo.2026.1789738

**Published:** 2026-06-08

**Authors:** Ye Liu, Lily Ng, Hong Liu, Douglas Forrest

**Affiliations:** National Institute of Diabetes and Digestive and Kidney Diseases, National Institutes of Health, Bethesda, MD, United States

**Keywords:** brain barrier, central nervous system, Dio3, neurodevelopment, thyroid hormone

## Abstract

Thyroid hormone is required for brain development but under constraint because its unregulated action can damage target tissues. Type 3 deiodinase, a thyroid hormone-degrading enzyme encoded by *Dio3*, constrains thyroid hormone exposure in the brain and has primarily been described in neuronal populations. However, detection is difficult because of low, transient expression, often in specialized cell populations. Here, using *Dio3*^Cre^-mediated cell labeling with a sensitive fluorescent reporter, we uncovered *Dio3* expression in non-neuronal cell types in blood-cerebrospinal fluid (CSF) and blood-brain barriers during embryonic and neonatal development in mice. These barriers control solute transport, suggesting that *Dio3* regulates thyroid hormone availability at cellular barriers between hormone-bearing fluids and the brain. *Dio3* expression was detected in the arachnoid barrier layer in the meninges, the membranes surrounding the brain, and in the epithelial layer of the choroid plexus, which regulates solute exchange with CSF. RNA analyses corroborated early *Dio3* expression in these tissues, beginning even before barrier function matures. *Dio3* expression was also detected in tanycytes lining the third ventricle in the mediobasal hypothalamus, in a restricted portion of the glia limitans at the ventral hypothalamus, and in pericytes at the neurovascular interface. These findings suggest a role for *Dio3* in regulating thyroid hormone availability at cellular interfaces with fluids that transport thyroid hormone in the immature brain.

## Introduction

Triiodothyronine (T3), the active form of thyroid hormone, promotes development of the mammalian nervous system, regulating events such as neuronal differentiation, migration and synaptogenesis (1, 2). Precise spatial and temporal control of T3 exposure within the nervous system is required because either insufficient or excessive thyroid hormone causes abnormalities (1, 3–6). A key factor that limits T3 signaling is type 3 deiodinase (DIO3), which depletes thyroid hormone by conversion of T3 and its precursor thyroxine (T4) into inactive metabolites (7, 8). This enzyme is critical for neurodevelopment as shown by *Dio3*-deficiency in mice which causes motor, behavioral (9, 10), gene expression (11), neuroendocrine (12–14) and sensory (15, 16) impairments.

The developmental expression of DIO3 is highly dynamic in the brain, as indicated by enzyme activity assays (9, 17–19), northern blot analyses, *in situ* hybridization analyses (20, 21) and Cre recombinase-mediated cell labeling (22). Typically, DIO3 activity peaks at immature stages and protects sensitive embryonic and neonatal tissues from over-stimulation by T3. As development progresses, expression often declines to low levels. To understand T3 functions in the brain requires identification of DIO3-expressing cell types but this has been difficult because of the transient, low expression levels. DIO3 is considered to act in target neurons, based on available mapping data (20, 22) and experimental studies (13, 23). In other systems, such as the testis and retina, indirect or paracrine-like mechanisms have also been proposed in which DIO3 expressed in certain cell types controls the availability of T3 for other, nearby target cell types (16, 24, 25). In the brain, the expression of *Dio3* in non-neuronal cell types including barrier tissues is poorly defined.

Barrier interfaces in the brain include the blood-cerebrospinal fluid (CSF) barrier and blood-brain barrier which regulate the exchange of essential nutrients, electrolytes and other solutes between fluids (CSF and blood) and neural tissues of the brain parenchyma (26, 27). Specialized cell layers connected by tight junctions prevent the free flow of solutes and regulate selective transport of necessary solutes through these barriers. From the bloodstream, T3 and T4 traverse the blood-brain barrier to gain access to neural tissues (28, 29) and also cross the blood-CSF barrier to enter CSF, which is considered another potential source of thyroid hormone for neural target cells (30, 31). Blood-CSF barriers include the arachnoid barrier layer in the meninges membranes around the brain and the epithelial layer of the choroid plexus that regulates secretion of CSF into the ventricles (26, 32). In early development, the immature meninges may also release signaling factors to promote differentiation of adjacent neocortical and calvarial cell types (33, 34). An inner CSF-brain barrier is formed by ependymal cells and tanycytes lining the ventricular surfaces of the brain. The blood-brain barrier consists of the endothelial cells of blood vessel walls connected by tight junctions, which are closely associated with supporting astrocytes and pericytes (27, 35).

In this study, we have taken advantage of a sensitive *Dio3*^Cre^ driver-reporter (22) to map *Dio3* expression in barrier tissues in mice at embryonic and neonatal stages when DIO3 activity generally peaks during brain development. This cell-labeling, anatomical approach identified *Dio3* expression in barrier cell types in their natural context in the immature meninges and choroid plexus, which we further supported by RNA expression analyses. The results also indicate *Dio3* expression in tanycytes and pericytes. These findings suggest a role for *Dio3* in regulating T3 availability at key barrier interfaces between blood, CSF and neural tissues during brain development.

## Materials and methods

### Mouse strains

The *Dio3*^Cre^ line carries a knockin Cre driver at the endogenous *Dio3* gene, as described previously (22). The Cre expression cassette is fused at the ATG initiation codon of the endogenous *Dio3* gene and does not disturb upstream sequences in the region of the *Dio3os* gene which resides on the opposite strand, upstream of *Dio3* (36). To visualize Cre-labeled cells and to avoid variations due to imprinting of the *Dio3* locus, male *Dio3*^Cre^ mice were crossed with female *Rosa26*^Ai6^ reporter mice (Ai6, Jackson laboratories, RRID: IMSR_JAX:007906) (37). These crosses generated *Dio3*^Cre/+^; *Rosa26*^Ai6/+^ mice for analysis of ZsGreen1 fluorescent protein expressed by the *Rosa26*^Ai6^ allele, upon Cre-mediated recombination. The *Dio3*^Cre^ and *Rosa26*^Ai6^ alleles were maintained in separate male and female parents, respectively, to prevent inadvertent germline activation of the Ai6 reporter allele. Male and female *Dio3*^Cre/+^ heterozygous offspring were analyzed with no sex-specific differences in Cre activity observed in tissues examined. Figures show representative results from at least 3 mice of the indicated genotype. The *Dio2*^CreERt2^ line carries a conditional CreERt2 driver knocked into the endogenous *Dio2* (type 2 deiodinase) gene and can activate a reporter (Ai6) in tissues where *Dio2* is expressed upon treatment with tamoxifen, as previously described (38). For analysis of the mediobasal hypothalamus, *Dio2*^CreERt2/+^;*Rosa26*^Ai6/+^ male mice were injected sub-cutaneously at postnatal days 10 and 11 (P10/P11) with 20 μL tamoxifen dissolved in corn oil at a concentration of 10 mg/mL (Sigma-Aldrich C-8267), then analyzed at 1 month of age, as described (38). The time of treatment with tamoxifen was based upon the reported postnatal rise of type 2 deiodinase activity in the rat hypothalamus (18) and was optimized for the *Dio2*^CreERt2^ mouse line by testing treatments at P3/P4, P12/P14 and P45, followed by analysis of tissue at approximately 6–8 weeks of age. The *Dio2*^CreERt2^ allele was genotyped as previously described (38). The *Dio3*^Cre^ allele was genotyped by PCR as described (22) with the following 3 primers: WT-F, 5′-CTA CAA CAA GGT GCA CCT GG-3′; Cre-F, 5′-AGC TGG TGG CTG GAC CAA TG-3′; cm-R, 5′-GAG TCT CAA GTT AGC CAG AC-3′, generating wild type (382 bp) and *Dio3*^Cre^ (278 bp) amplicons. All procedures were conducted in accordance with protocols and guidelines approved by the Animal Care and Use Committee of the National Institute of Diabetes and Digestive and Kidney Diseases (NIDDK) at the National Institutes of Health (NIH).

### Immunostaining and image acquisition

Tissues were fixed in 2% paraformaldehyde (PFA) for 4 hours, followed by cryoprotection in 30% sucrose overnight. Tissues were embedded in OCT compound (Tissue-Tek) and cryosectioned at a thickness of 12 μm. For immunostaining, sections were immersed in 5% donkey serum in PBST (phosphate-buffered saline with 0.01% Triton X-100) for 1 hr at room temperature, incubated with primary antibodies (or lectin) overnight at 4 °C, then incubated with secondary antibodies for 1 hr at room temperature. Sections were counter-stained with Hoechst (33342, Invtrogen, H3569) or DAPI (4’,6-diamidino-2-phenylindole, Vectashield H1800, Vector Laboratories). Primary antibodies, secondary antibodies and lectin reagents are listed in **Table 1**. Fluorescent images were acquired using a Nikon Ti2 inverted widefield microscope or a Nikon-SoRa spinning disk confocal microscope. Images were processed using ImageJ software (RRID: SCR_003070).

**Table 1:** Antibodies and lectin reagents.

Antibody or reagent	Source	Catalogue	Type	Clonal	Dilution	RRID
Primary antibodies						
Collagen IV	Abcam	Ab6586	rabbit	polyclonal	1:300	AB_305584
CRABP2	Proteintech	10225-1-AP	rabbit	polyclonal	1:200	AB_2085455
E-Cadherin	R and D Systems	AF748	goat	polyclonal	1:200	AB_355568
Ezrin(3C12)	Santa Cruz	Sc-58758	mouse	monoclonal	1:50	AB_783303
GFAP	Abcam	Ab7260	rabbit	polyclonal	1:500	AB_305808
NeuN	Millipore	ABN78	rabbit	polyclonal	1:400	AB_10807945
NG2	Invitrogen	MA5-24247	rat	monoclonal	1:200	AB_2606388
PDGFRb	Thermofisher	14-1402-82	rat	monoclonal	1:250	AB_467493
Phospho-PDGFRb (Tyr716)	Santa Cruz	sc-365464	mouse	monoclonal	1:60	AB_10847085
SOX2	Santa Cruz	Sc-17320	goat	polyclonal	1:300	AB_2286684
Secondary antibodies						
Alexa Fluor 568 anti-rabbit IgG	Thermofisher	A-10042	donkey	polyclonal	1:300	AB_2534017
Alexa Fluor 647 anti-rabbit IgG	Thermofisher	A-31573	donkey	polyclonal	1:300	AB_2536183
Alexa Fluor 568 anti-rat IgG	Thermofisher	A-78946	donkey	polyclonal	1:300	AB_2910653
Alexa Fluor 647 anti-goat IgG	Thermofisher	A-21447	donkey	polyclonal	1:300	AB_2535864
Alexa Fluor 568 anti-mouse IgG	Thermofisher	A-10037	donkey	polyclonal	1:300	AB_11180865
Lectin						
Alexa Fluor 568 isolectin GS-IB4	Thermofisher	121412	N/A	N/A	1:400	AB_2314662

### Tissue dissection and RNA extraction

The brain of mouse embryos or pups (E13.5 to P4, wild type, mixed C57BL/6J x 129/sv/J background) of either sex was dissected in ice-cold PBS under a stereomicroscope with the leptomeninges kept intact on the brain surface, as described (39). The telecephalic hemispheres were separated and leptomeninges carefully isolated. To dissect the choroid plexus from the 4^th^ ventricle, the cerebellum was separated from the brainstem to expose the 4^th^ ventricular cavity, then the choroid plexus was dissected from surrounding tissue, based on distinct morphology. Each sample of leptomeninges was a pooled from 3 embryos or pups and each sample of choroid plexus was pooled from 4 embryos or pups. Total RNA was extracted from collected tissues using RNeasy Plus mini kit (Qiagen, Cat #74136) following the manufacturer’s protocol.

### Quantitative real-time PCR analysis of gene expression

Total RNA was used for cDNA synthesis using oligo(dT) primers and Superscript IV Reverse Transcriptase (Thermo Fisher, Cat #18091050). Quantitative PCR reaction was performed with FastStart Universal SYBR Green Mix (Millipore Sigma, Cat #4913914001) on a StepOne or QuantStudio 3 machine, and data were analyzed using manufacturer’s software (Thermo Fisher). Relative gene expression levels were quantified using the 2^−ΔΔCT method, with *Hprt* as the internal control, as described (40). Primer pairs were as follows: Hprt-F, 5’-TAC CTC ACT GCT TTC CGG AG-3’, Hprt-R, 5’-ATC GCT AAT CAC GAC GCT GG-3’; Dio3-394R, 5’-CCT CAT GGG CTT GCT TGA AG-3’, Dio3-250F, GTA GAG CTC AAC AGT GAA GG-3’.

### RNA-seq library construction and data analysis

RNA prepared from leptomeningeal and choroid plexus samples as described above, was used to construct libraries with a SMARTer stranded total RNA sample prep kit (Takara #634875), following the manufacturer’s instructions. Libraries were sequenced on an Illumina NovaSeq X apparatus at the NHLBI Genomics Facility at NIH. Between 10 and 20 million pair-end (50 base) reads/per library were collected, converted by bcl2fastq (version 2) into fastq files and analyzed for gene expression using Kallisto v0.51.1 (https://github.com/pachterlab/kallisto/releases) with units given as TPM (transcripts per million read counts) according to quantification of reads on a GRCm38/mm10 index.

*Heatmap analysis*: Gene expression values from different samples were log2-transformed, followed by scaling of data to calculate the z-score for each gene across samples within the comparison group, as described (41). Final output results were visualized as a heatmap created using heatmap.2 function in RStudio (2022.07.2 Build 576).

RNA-seq datasets that we generated for different developmental stages of the leptomeninges and choroid plexus are available in the GEO database (GSE315778).

We analyzed a publicly available, spatial transcriptomic resource of adult mouse brain datasets at the Allen Institute (adult mouse brain MERSCOPE v1 – imputed genes and reconstructed coordinates, https://brain-map.org/bkp#2d-brain).

### Statistical analysis

Data for qPCR analyses are presented as means ± standard deviation (SD). Statistical analysis was performed using one-way ANOVA followed by Tukey’s *post-hoc* test for multiple pairwise comparisons; *P* < 0.05 was considered significant. Analyses were performed using GraphPad Prism version 10.1.1.

## Results

### *Dio3* expression in the arachnoid barrier layer of the meninges

To facilitate sensitive detection of *Dio3*-expressing cell types in their natural context in brain barrier tissues, we used a *Dio3*^Cre^ knock-in driver that expresses Cre recombinase from the endogenous *Dio3* gene (22). In tissues where *Dio3* is expressed, Cre activates expression of ZsGreen1 (ZsG) fluorescent protein from a *Rosa26*^Ai6^ (Ai6) reporter gene (37). The expression pattern reflects endogenous *Dio3* expression (3, 9, 20), as shown previously for specific neuronal populations in the brain, retinal progenitor cells and sensory ganglia (22). For monitoring expression of *Dio3*, a gene with peak expression at embryonic stages (17, 18), the *Dio3*^Cre^ driver allele has an advantage in labeling cells following the developmental onset of *Dio3* expression once Cre recombinase protein attains a threshold level that can activate the reporter gene. This approach creates a permanent ZsG+ signal in labeled cells regardless of subsequent decreases in *Dio3* expression levels in these cells.

We focused on embryonic and neonatal stages since DIO3 enzyme activity is known to peak during early development of diverse brain regions (9, 17, 18). Analyses were performed on heterozygous *Dio3*^Cre/+^ progeny (i.e., *Dio3*^Cre/+^;*Rosa26*^Ai6/+^ abbreviated as Dio3Cre;Ai6). The *Dio3*^Cre^ allele was derived from the male parent to avoid variations in expression due to differential imprinting of *Dio3* alleles of paternal or maternal origin (42).

**Figure 1:**
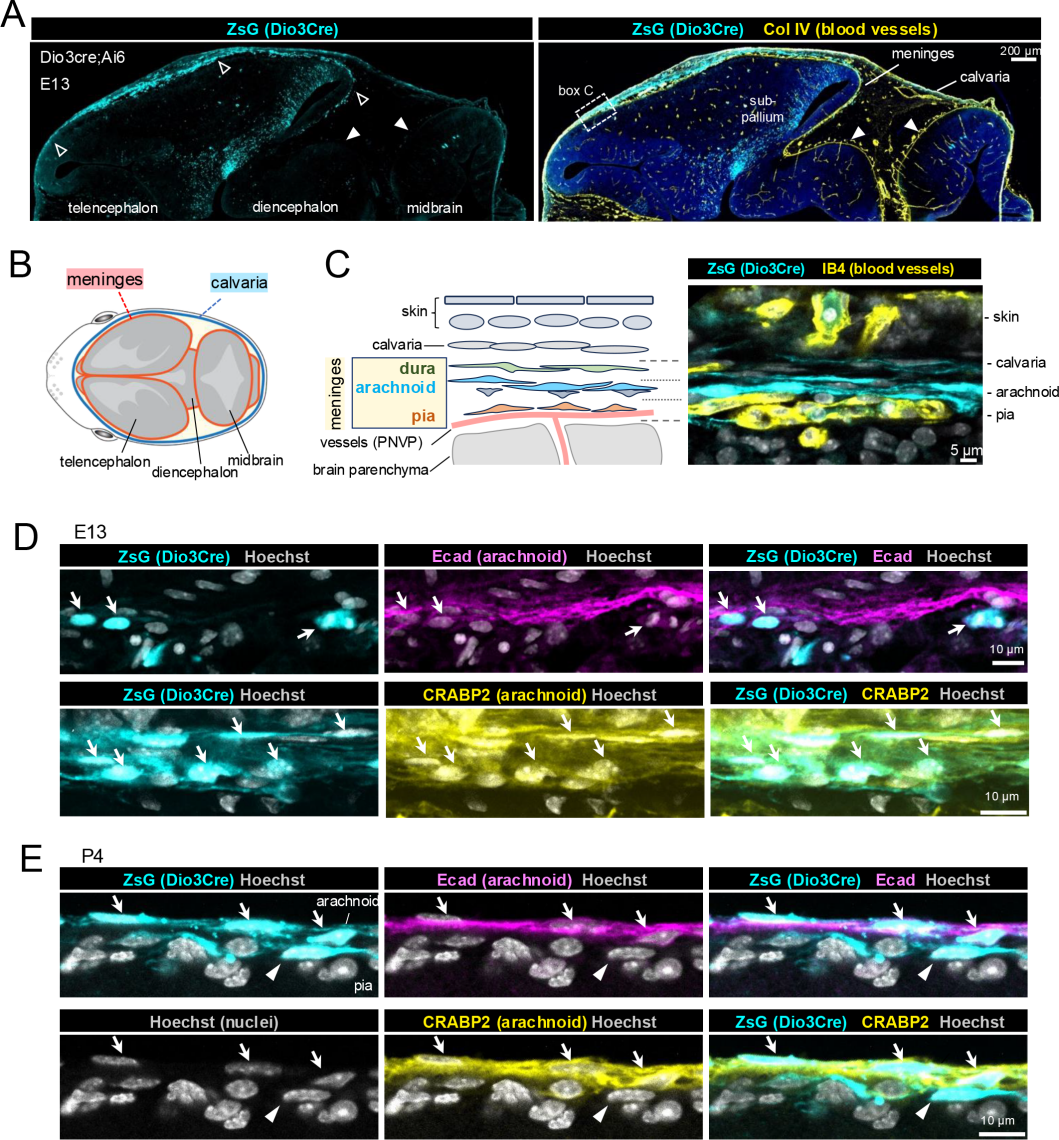
*Dio3* expression in the meninges identified using *Dio3*^Cre^-mediated labeling. **(A)** An overview, transverse section showing *Dio3* expression in the meninges (open arrowheads) detected as *Dio3*^Cre^-activated ZsG signals induced from a *Rosa26*^Ai6^ reporter gene, at embryonic day 13 (E13) (n = 3, male and female mouse embryos). This cryosection shows one hemisphere of the brain in the plane indicated in panel **(B)** Col IV labels the vasculature around all meningeal regions and within the brain. Note ZsG+ signals in meningeal regions in the forebrain (open arrowheads) but minimal signals around mid- and hindbrain regions (white-fill arrowheads). **(B)** Diagram of a transverse view of an embryonic mouse brain, showing meninges locations as a red line. **(C)** *Left*, diagram of cell types in meninges and adjacent tissue. *Right*, High-magnification view of the meninges at postnatal day 4 showing *Dio3* expression (ZsG) predominantly in the arachnoid layer, external to IB4+ vessels of the pia. Cryosection of a telencephalic area, approximately in the location of “box C” in panel **(A) (D)** *Dio3* (ZsG) expression in the immature meninges at E13. Each row shows a different field of view identifying ZsG+ arachnoid barrier cells by co-localization with E-cadherin and CRABP2 (white arrows). Scale bar in the right panel applies to all images in the same row. **(E)** *Dio3* (ZsG) expression in the arachnoid barrier layer at a more mature, neonatal stage (P4). Both rows show the same field of view identifying ZsG+ arachnoid barrier cells by co-localization with E-cadherin and CRABP2 (white arrows). A subset of ZsG+ cells was detected in the pia mater (up-pointing arrowhead). Scale bar in bottom right panel applies to all panels in **(E)** Col IV, Collagen IV; CRABP2, cellular retinoic acid binding protein 2; Ecad, E-cadherin; IB4, isolectin B4; ZsG, ZsGreen.

At embryonic day 13 (E13), ZsG+ cells were detected in the nascent meninges around the brain, in cranial mesenchyme and calvaria, and as reported previously, in formative brain regions such as the subpallium that gives rise to parts of the amygdala and other brain areas (20, 22) (**Figures 1A, B**). The meninges consist of 3 main layers: the dura mater (outer fibrous layer), arachnoid mater (middle, epithelial-like layer of arachnoid barrier cells) and pia mater (inner, vascularized connective tissue layer with vessels that enter the brain). The arachnoid and pial layers together constitute the leptomeninges (**Figure 1C**). High magnification views at E13 identified ZsG+ cells in the nascent arachnoid barrier layer, as indicated by the location of these cells immediately external to vessels of the pial perineural vascular plexus (PNVP, labeled by IB4 endothelial marker) (**Figure 1C**) and by co-localization with the arachnoid marker E-cadherin (**Figure 1D**). A subset of ZsG+ cells was observed in the PNVP region that may represent fibroblasts in the formative pia (43) and a few ZsG+ cells were seen in the skin, consistent with *Dio3* expression during development of the epidermis (44) (**Figure 1C**). In the meninges, ZsG was co-localized with CRABP2, a marker of arachnoid cells and of more externally located dural fibroblasts at E13 (43). These results indicate *Dio3* (ZsG) expression in the formative arachnoid layer and in a subset of cells in the nascent pial layer at embryonic stages.

At E13, the meninges cell layers are more diffusely organized than at mature ages. Therefore, we examined the meninges at postnatal day 4 (P4), when the tissue is more defined, which confirmed the arachnoid identity of ZsG+ cells by co-localization with E-cadherin and CRABP2 (**Figure 1E**). A subset of ZsG+ cells were again located internally relative to the arachnoid layer that may represent pial fibroblasts (upward pointing arrowhead, **Figure 1E**).

*Dio3* expression in the meninges suggests a possible role in regulating T3 and T4 during transport through the arachnoid barrier to the subarachnoid space (**Figure 1C**). However, as there is minimal subarachnoid space associated with the immature meninges at E13 and the meninges may not form tight junctions until later embryonic stages (45), other functions are possible for *Dio3* in limiting T3 signaling in this local cellular environment at immature stages.

### Regional heterogeneity of *Dio3* expression in the meninges

**Figure 2:**
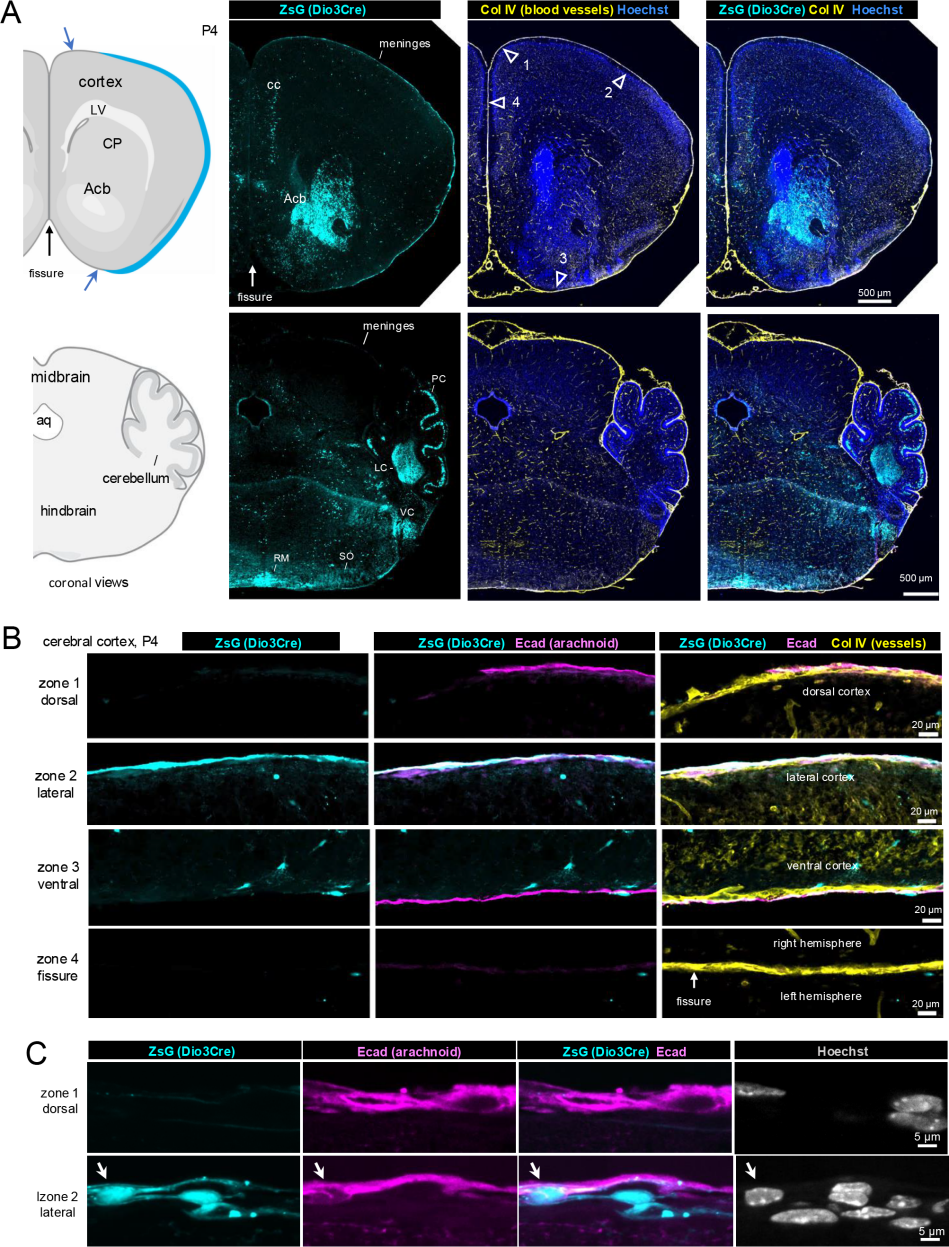
Regional variations of *Dio3* expression in the meninges around the immature brain. **(A)** *Upper row*, *Dio3* (ZsG) expression is prominent in the meninges in the lateral telencephalon. Signals are sparser in dorsal/ventral areas and undetected in the central fissure. *Lower row*, in more caudal brain regions, weak or no ZsG signals are detected in the meninges in the midbrain, cerebellum and hindbrain. Col IV, a vascular marker, reveals meninges in all regions. Scale bar in top right panel is the same in all panels in **(A)** Reference diagrams for coronal views are on the *left*. Acb, Accumbens nucleus; aq, aqueduct of the midbrain; cc, cingulate cortex; CP, caudoputamen; Lc, lateral cerebellar nucleus; LV, lateral ventricle; PC, Purkinje cells; RM Raphe magnus nucleus; SO, superior olive; VC, ventral cochlea nucleus. **(B)** Magnified views of selected meningeal zones around the cerebral cortex showing *Dio3* (ZsG) expression restricted to the lateral and ventral zones with minimal expression in other zones. Zones 1–4 are indicated in panel *A*. ZsG signals are mainly in zone 2 (lateral). E-cadherin (Ecad) is a marker of lateral, ventral and some dorsal areas but is not all meningeal areas. Col IV indicates blood vessels in all meningeal regions. Scale bars on the *right* apply to all panels in a given row. **(C)** High magnification views showing *Dio3* (ZsG) expression in lateral but not dorsal areas of the meningeal arachnoid layer around the cortex. Scale bar applies to all panels. Arrows indicate *Dio3* (ZsG)-expressing cells. Scale bars on the *right* apply to all panels in a given row.

The transverse overview sections of the embryonic brain in **Figure 1A** indicated regional restriction of *Dio3* expression (ZsG) in the meninges. ZsG signals were detected around the forebrain (open arrowheads) but signals were weak or undetectable in midbrain and hindbrain regions (white fill arrowheads). The outline of the meninges, indicated by collagen IV, a blood vessel marker, extended around all brain regions unlike the restricted pattern of ZsG signals. We investigated regional heterogeneity of *Dio3* expression in the leptomeninges in more detail in coronal sections of the neonatal brain at P4 (**Figure 2A**). Prominent *Dio3* (ZsG) signals were observed in the meninges around the forebrain, mainly in lateral regions (e.g., zone 2 in **Figure 2A**). Signals were sparser in dorsal and ventral regions towards the midline (zones 1 and 3) and were absent in the central, longitudinal fissure that separates the cerebral hemispheres (zone 4). Along the rostral-caudal axis, ZsG signals were sparse or undetected in the meninges of the midbrain and hindbrain (**Figure 2A**, lower panels) compared to the forebrain (upper panels). In **Figure 2B**, higher-magnification views of the meninges around selected forebrain zones (denoted by arrowheads in **Figure 2A**) confirmed the presence of ZsG signals in lateral regions (zone 2), minimal signals in dorsal or ventral regions towards the fissure (zones 1 and 3) and no detectable signal in the fissure itself (zone 4). Where expressed (i.e., in more lateral zones), ZsG signals were detected in the arachnoid barrier layer, indicated by co-localization with E-cadherin as shown in medium-magnification (**Figure 2B**) and high-magnification (**Figure 2C**) views. This regional restriction of expression in the meninges is consistent with the pattern of *Dio3* (ZsG) signals that can be observed in a series of coronal brain sections at P1 in a previous report (22). These results indicate regional variations of *Dio3* expression in the arachnoid layer that may contribute to spatial differences in the regulation of T3 availability during brain development.

### *Dio3* expression in the choroid plexus epithelium during development

**Figure 3:**
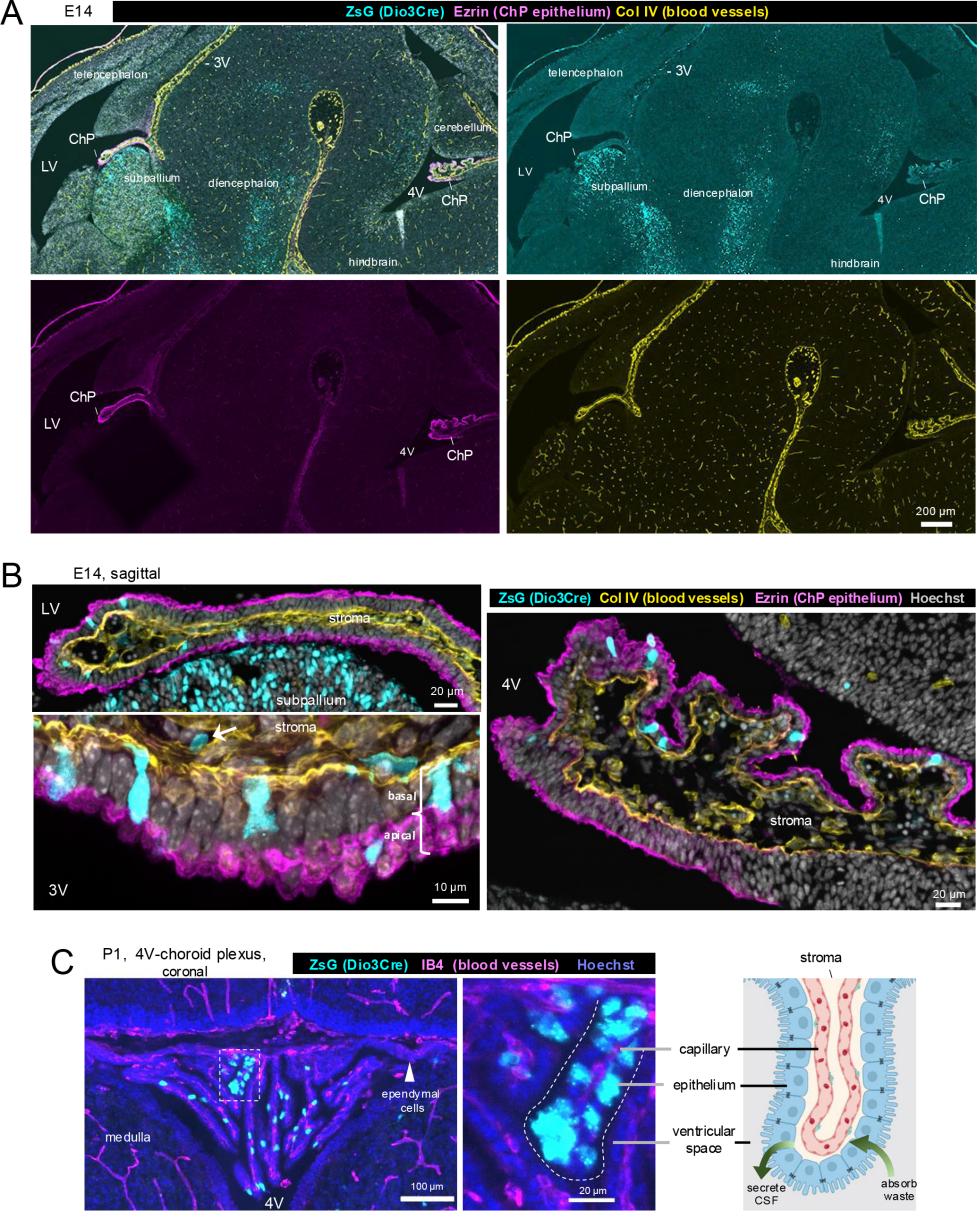
*Dio3* expression in the immature choroid plexus. **(A)** Sagittal section of the brain at E14 showing the choroid plexus (ChP) in the lateral ventricle (LV) and 4^th^ ventricle (4V). Images of this section show *Dio3* expression (ZsG), choroid plexus epithelial marker, Ezrin and blood vessel marker, Col IV (collagen IV). Scale bar on bottom right panel applies to all panels. **(B)** High magnification views of ZsG+ cells in the choroid plexus in telencephalic (lateral ventricle, LV), diencephalic (3^rd^ ventricle, 3V) and hindbrain (4^th^ ventricle, 4V) regions at E14. ZsG+ cells reside in the choroid plexus epithelium, identified with Ezrin, a marker of the apical side of these epithelial cells, facing the cerebrospinal fluid. ZsG signals are also detected in a few stromal cell (example indicated by white arrow) in the 3V choroid plexus. **(C)** Representative coronal section of the choroid plexus in the 4V at P1. ZsG+ cells are in choroid plexus epithelial cells but are rarely detected in the IB4+ stromal/capillary core of the choroid plexus or in ependymal cells. *Right*: diagram of cell layers of the choroid plexus relative to the cerebrospinal fluid and capillaries in the stromal core.

The choroid plexus consists of an epithelial cell monolayer that forms villi around a core of capillaries and stromal connective tissue (46). Choroid plexus structures are found in the lateral, 3^rd^ and 4^th^ ventricles of the brain and regulate both the production and composition of the CSF. The choroid plexus also synthesizes transthyretin, a carrier protein involved in transfer of thyroid hormone from the blood to CSF (47). The location of the choroid plexus in the lateral and 4^th^ ventricles is revealed by the epithelial marker Ezrin in sagittal overview sections at E14 in **Figure 3A** (the choroid plexus in the 3^rd^ ventricle is not evident in this plane of view). High magnification analyses at E14 identified ZsG signals (*Dio3* expression) in the choroid plexus in the lateral, 3^rd^ and 4^th^ ventricles as shown (**Figure 3B**). ZsG was primarily detected in the columnar epithelial cells of the choroid plexus epithelium as indicated by co-staining for Ezrin, a marker of the apical membrane of these cells (**Figure 3B**). Occasional ZsG+ cells were detected in the vascular stromal layer, identified by the stromal marker Col IV (**Figure 3B**). At P1, ZsG signals were confirmed in choroid plexus epithelial cells, when the tissue is more mature, as shown in the example of the 4^th^ ventricle in **Figure 3C**. ZsG+ cells were undetected in the ependymal layer that is contiguous with the choroid plexus epithelium. The finding of *Dio3* expression in the choroid plexus epithelium suggests a possible role in modulating T3 and T4 content in the CSF during neurodevelopment.

The ZsG+ cells detected in the choroid plexus epithelium in each location were relatively sparse, which may be explained by low and developmentally declining *Dio3* expression. Low expression of the driver gene may limit the proportion of cells that attain sufficient Cre levels that can induce ZsG from the Ai6 reporter.

### *Dio3* RNA expression in the meninges and choroid plexus

**Figure 4:**
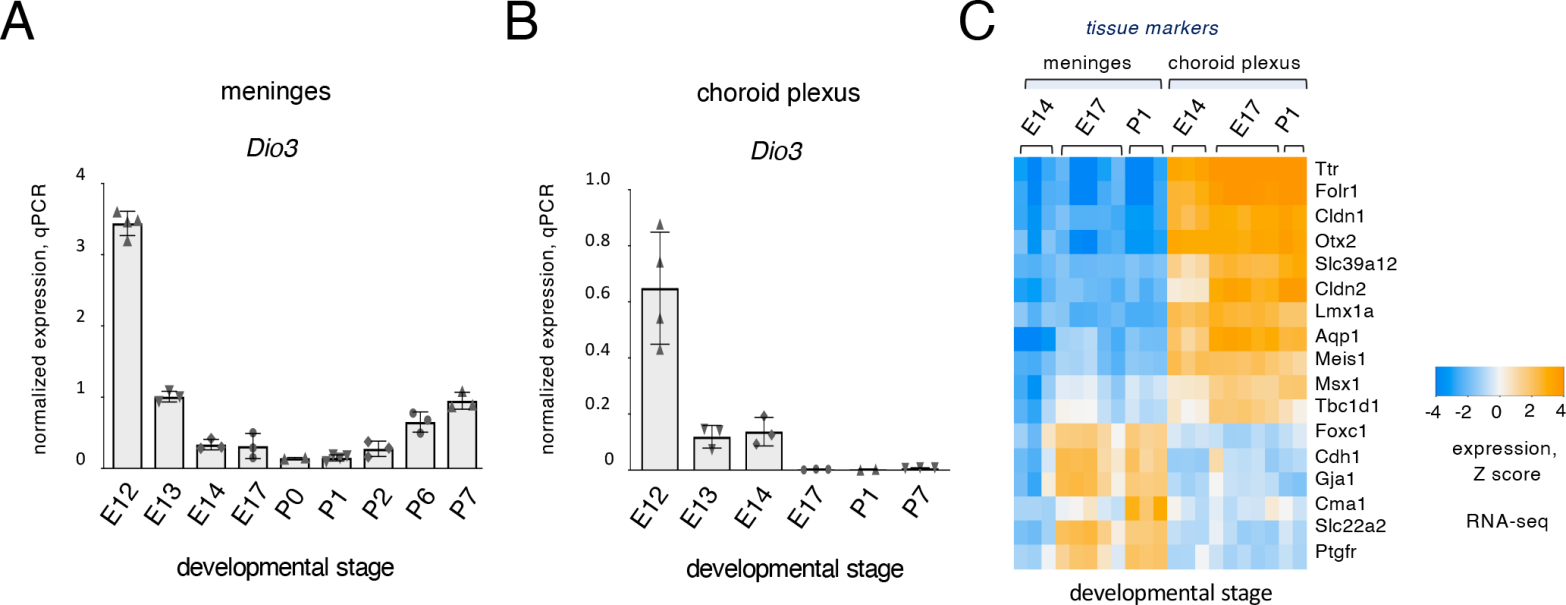
Developmental expression of *Dio3* RNA in the meninges and choroid plexus. **(A, B)** Dynamic *Dio3* expression profile with a peak in early development of the leptomeninges **(A)** and choroid plexus **(B)**, determined by qPCR analysis. In both tissues, *Dio3* peaks around E12 then declines by neonatal stages. In the leptomeninges but not choroid plexus, *Dio3* increases modestly again at postnatal stages up to P7. Means ± SD; n = 3 or 4 samples (wild type, each representing 3–4 pooled mice at each stage). **(C)** Heatmap from RNA-sequencing analysis showing *s*pecificity of the micro-dissected tissues with marker gene expression for leptomeninges and choroid plexus during development (n = 3–5 per age, except choroid plexus at P1, n = 2).

To corroborate *Dio3* expression detected in the meninges and choroid plexus by Cre-mediated labeling, we used quantitative PCR (qPCR) analyses of micro-dissected tissues over an extended developmental time course (E12 to P7) in wild type mice (**Figure 4**). The goals were two-fold: (i) to confirm expression of *Dio3* in the tissue by an independent approach; and (ii) to indicate the developmental profile of *Dio3* expression because temporal changes cannot be revealed by Cre-mediated labeling which creates permanent ZsG signals regardless of later changes in expression of the *Dio3* driver gene. In the leptomeninges isolated from the telencephalon, qPCR revealed an early peak of *Dio3* expression at E12, then steep decline to E17 (**Figure 4A**), consistent with the identification of positive cells using the *Dio3*^Cre^ driver as early as E13 (**Figure 1**). From E12 to E14, *Dio3* transcript levels declined approximately 10-fold from a mean level of 3.44 to 0.33 (P < 0.001) and continued to decline to neonatal stages (at P1, mean = 0.15)(P < 0.001). The early peak of *Dio3* RNA expression in the forebrain meninges parallels the early peak of type 3 deiodinase enzyme activity previously reported for rodent cerebral tissue (17, 18). Interestingly, a later, modest rise of *Dio3* expression was detected by qPCR in the first postnatal week (P < 0.001, for P7 compared to P1) suggesting possible functions at postnatal ages.

We also validated *Dio3* expression by qPCR analysis over this same period for the choroid plexus isolated from the 4^th^ ventricle (**Figure 4B**). An early peak at E12 followed by a marked decline suggested functions at immature stages. *Dio3* transcript levels decreased approximately 5-fold from E12 to E14 (P < 0.001), then decreased to almost undetectable levels at later embryonic and neonatal stages. The generally lower values for *Dio3* RNA expression in the choroid plexus than the leptomeninges were in accord with the sparser cell labeling detected in the choroid plexus using the *Dio3*^Cre^ driver. The steep decline of *Dio3* transcript levels in the choroid plexus during embryonic development suggests another reason why only sparse labeling was achieved with the *Dio3*^Cre^ driver. For Cre protein to attain a level that can activate the reporter, an adequate period of developmental time is required. This level may be unattainable in some choroid plexus cells if expression of the *Dio3* driver drops soon after the initial peak of expression.

As the embryonic meninges and choroid plexus yield very small pieces of tissue, we established the specificity of these microscopically-dissected samples by analysis of known markers in RNA-seq datasets we generated for each tissue at selected developmental stages (**Figure 4C**). The results indicated high specificity with the meninges expressing expected markers, such as *Foxc1* and *Ptgfr* whereas the choroid plexus expressed its own characteristic markers, including *Ttr* and *Cldn1*.

### *Dio3* expression in tanycytes in the hypothalamus

**Figure 5:**
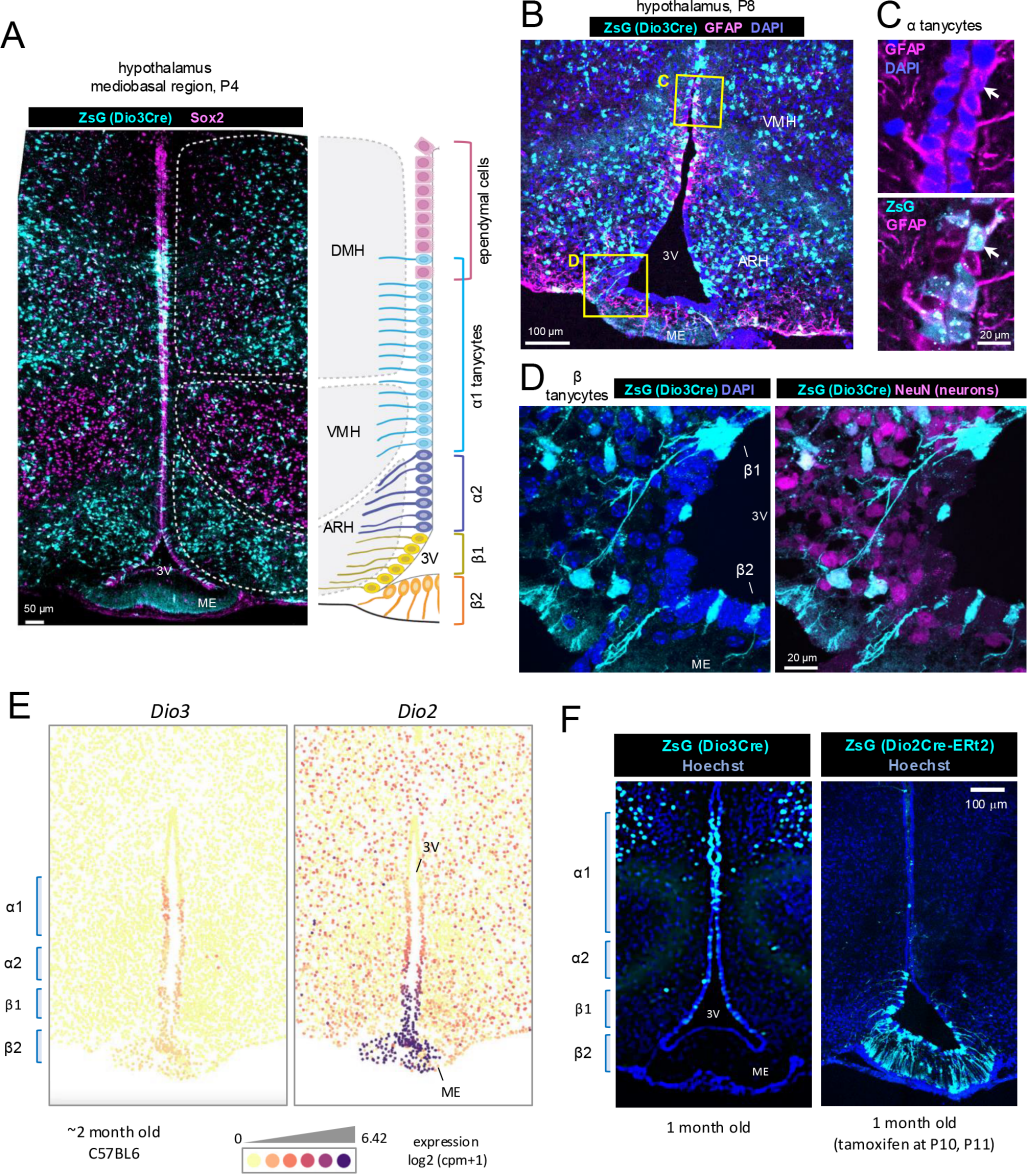
*Dio3* expression in tanycytes in the mediobasal hypothalamus. **(A)** *Left*, Overview showing *Dio3* expression (ZsG) in tanycytes of the 3^rd^ ventricle (3V) in the mediobasal hypothalamus (MBH) at P4; coronal section. *Right*: diagram illustrating tanycyte subtypes (α1, α2, β1, β2) with processes extending into hypothalamic dorsomedial nucleus (DMH), ventromedial nucleus (VMH), arcuate nucleus (ARH) and median eminence (ME). Sox2 labels nuclei of ependymal cells, tanycytes and neuronal subsets. **(B)** Higher magnification overview of the MBH indicating approximate areas of magnified views in C, D. **(C, D)** Magnified views of α- **(C)** and β-tanycytes **(D)** in a coronal section at P8. ZsG+ α-tanycytes extend GFAP+ processes into the VMH (arrow) **(C)** whereas β1-tanycytes extend processes into the arcuate nucleus and β2-tanycytes extend processes into the median eminence **(D)**. Hypothalamic areas are stained with NeuN, to indicate neuronal cell bodies. Nuclei counterstained with DAPI. In C, scale bar applies to both panels. In D, scale bar applies to both panels. **(E)** Comparative analysis of *Dio3* and *Dio2* transcripts detected in the 3^rd^ ventricle area of the MBH in adult mice in a spatial transcriptomics resource at the Brain Knowledge Platform, MERSCOPE v1 (adult mouse brain – imputed genes and reconstructed coordinates, https://brain-map.org/bkp#2d-brain). A relative expression scale generated by the Brain Knowledge Platform is below the images. Both images are derived from the same representative section (#C57BL6J-638850.36). **(F)** Comparison of *Dio3* and *Dio2* expression (ZsG signals) in tanycyte subtypes in the hypothalamic region in one month old mice revealed using *Dio3*^Cre^ and *Dio2*^CreERt2^ driver alleles, each in combination with Ai6 reporter (*Rosa26*^Ai6/+^), as described in the Results and Methods sections. Cre-mediated labeling using the conditional *Dio3*^CreERt2^ driver was achieved following treatment with tamoxifen at postnatal days 10 and 11. Scale bar in right panel applies to both panels. Note the similarity of expression patterns detected by both methods in panels **(E, F)**.

Tanycytes line the ventrolateral walls of the 3^rd^ ventricle and extend processes into several hypothalamic areas. Tanycytes are specialized for communication and transport of solutes between CSF, neurons and blood vessels in the hypothalamic parenchyma (48). Subtypes of tanyctes have been described according to their position along the ventricular wall relative to different hypothalamic regions: α1 types reside near the dorsomedial (DMH) and ventromedial (VMH) nuclei, α2 types near the arcuate nucleus (ARH) and β1 types and β2 types near the ARH and median eminence (ME) (49, 50) (**Figure 5A**). DIO3 enzyme has a known peak of activity in hypothalamic tissue at late embryonic and neonatal stages, then a decline at juvenile ages to relatively low levels at adult ages in rodents (12, 18).

Using *Dio3*^Cre^-mediated labeling, we located *Dio3* expression (ZsG) in tanyctes in the 3^rd^ ventricle wall at neonatal stages. A tanycyte identity was confirmed by co-localization with Sox2 a marker of tanycytes and ependymal cells (**Figure 5A**). Nearby ependymal cells in the upper dorsomedial region lacked ZsG signals. *Dio3* expression (ZsG) was prominent in α1 tanycytes but was sparser in α2, β1 and β2 tanyctes. As tanycytes become terminally differentiated, they express glial fibrillary acidic protein (GFAP). At P8, a tanycyte identity for ZsG+ cells was confirmed by co-localization with GFAP, which revealed characteristic GFAP+ processes extending into hypothalamic nuclei (**Figure 5B**). **Figure 5C** shows ZsG+/GFAP+ tanycytes (α type) at higher magnification. Double-staining with the NeuN neuronal marker (**Figure 5D**), revealed the processes of ZsG+ tanycytes (β1 and β2 types) extending between neuron cell bodies towards the basal region of the ME. The finding of *Dio3* expression in tanycytes, including both α and β subtypes, suggests a role in regulation of T3 availability at CSF–brain interfaces in hypothalamic development. As reported previously (22), *Dio3* (ZsG) expression was also observed in neurons in several hypothalamic centers at postnatal stages (e.g., VMH, DMH, ARH, **Figure 5A**).

To corroborate the expression of *Dio3* (ZsG) in tanyctes detected by Cre-mediated labeling, we investigated *Dio3* transcript expression in a spatial transcriptomics resource for adult mouse brain (51) (**Figure 5E**) (Brain Knowledge Platform, MERSCOPE v1, Imputed genes and reconstructed coordinates, https://brain-map.org/bkp#2d-brain). *Dio3* transcripts were detected in a pattern closely resembling that revealed by *Dio3*^Cre^-mediated labeling (**Figure 5E**). *Dio3* transcript signals were generally low but were stronger in the α1/α2-tanycte region and weaker in β1/β2-tanycyte regions. These findings also indicate that *Dio3* expression in tanycytes in the 3^rd^ ventricle is maintained into mature ages (2 month adult stage).

The T3-amplifying enzyme, type 2 deiodinase (DIO2) also contributes to the regulation of T3 functions in the hypothalamus (52) and DIO2 enzyme activity levels rise during postnatal hypothalamic development in the rat (18). Therefore, we investigated the possible combined roles of DIO2 (T3-amplifying) and DIO3 (T3-inactivating) enzymes in tanycytes by analysis of *Dio2* and *Dio3* transcript expression using the MERSCOPE v1 resource (**Figure 5**). *Dio2* transcripts were prominent in β tanycytes in ventral regions and the ME and were detected at lower levels in α tanycytes in dorsal regions of the 3^rd^ ventricle. The results yield fine cellular detail that extends data from *in situ* hybridization of rat hypothalamus (53). Together, the findings indicate that both deiodinase genes are expressed but in opposing gradients with *Dio3* prominent in dorsal regions and *Dio2* in ventral regions. The results also suggest overlapping expression of both *Dio3* and *Dio2* genes in tanycytes but in differing proportions along the dorsal-ventral axis.

To correlate the spatial transcriptomics analysis with Cre-mediated labeling, **Figure 5F** shows ZsG signals at 1 month of age in the hypothalamic 3^rd^ ventricle and ME detected using knockin Cre drivers for *Dio3* and *Dio2* (38) genes. The pattern of *Dio3*^Cre^-mediated labeling at 1 month of age closely resembled that at P4 (see **Figure 5A**) and correlates with the spatial transcriptomics pattern for *Dio3* at 2 months of age (**Figure 5E**). The pattern of *Dio2*^CreERt2^-mediated labeling correlates with the *Dio2* transcript expression pattern in **Figure 5E**. Cre-mediated labeling using the conditional *Dio2*^CreERt2^ driver required prior treatment with tamoxifen at optimized postnatal ages (P10, P11) when *Dio2* expression rises in the rodent hypothalamus (18), as described in the Methods section.

### *Dio3* expression in a region of the glia limitans

**Figure 6:**
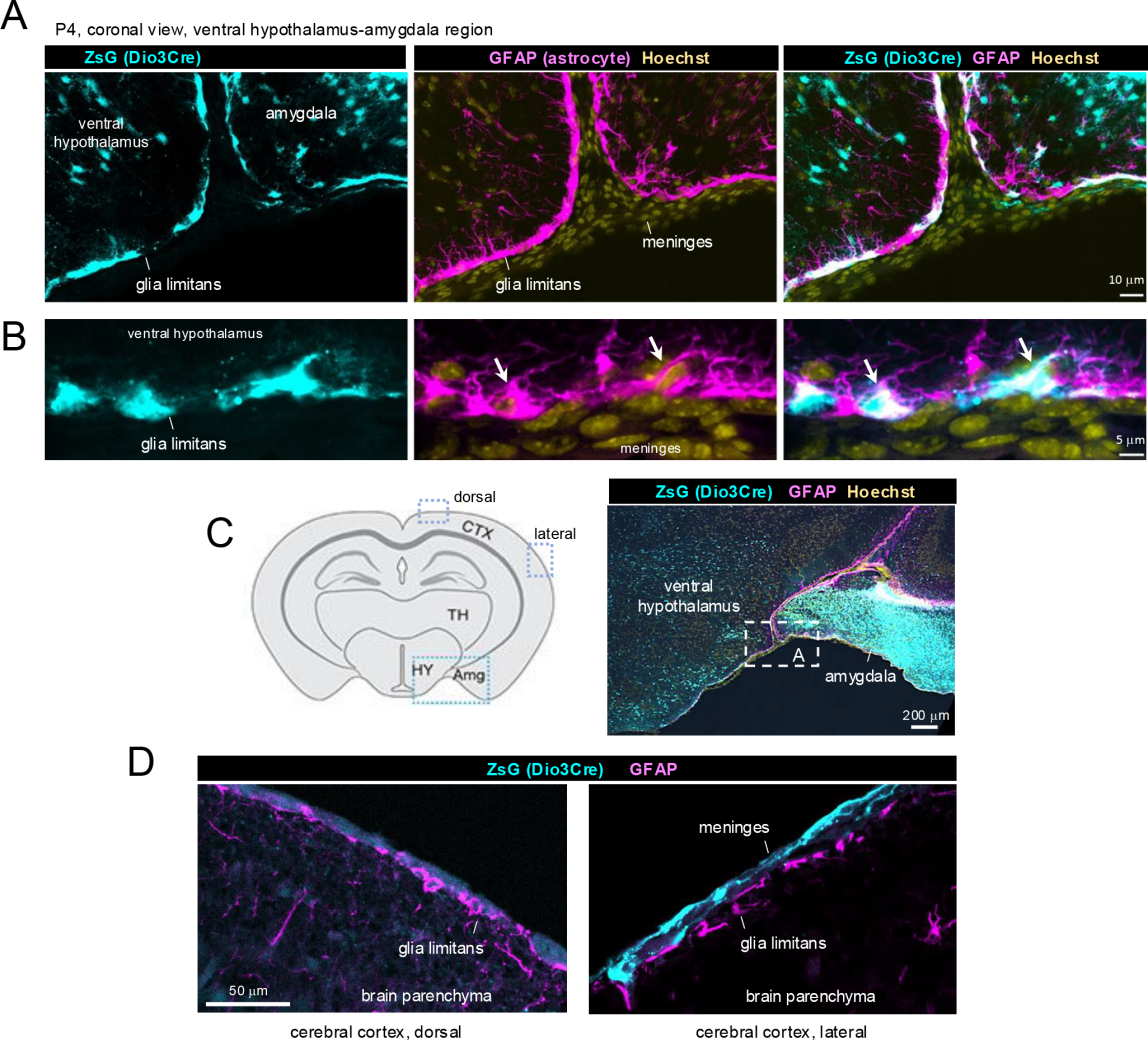
*Dio3* expression in a region of the glia limitans. **(A)** *Dio3* expression (ZsG) in astrocytes of the glia limitans at the boundary of the ventral hypothalamus near the border of the amygdala. Coronal views show co-localization of ZsG with astrocyte marker GFAP (glial fibrillary acidic protein). Scale bar on *right* applies to all panels in **(A) (B)** Magnified views of areas in panel **(A)** Scale bar on *right* applies to all panels in **(B)**. **(C)**. *Left*, diagram of coronal view of the brain with boxed area “A” indicating the approximate areas shown in panel **(A)** Boxed areas annotated as “dorsal” and “ventral” indicate approximate areas shown in panel **(D)** *Right*, Coronal section showing an overview of the brain regions around box “A” with imaging for *Dio3* (ZsG) and astrocytes (GFAP). *Dio3* expression is prominent in the nearby areas of the amygdala and ventral hypothalamus, as expected (see Results text). CTX, cortex; TH, thalamus; HY, hypothalamus. **(D)** Magnified views of the glia limitans and meninges in dorsal and lateral areas around the cerebral cortex at P4. ZsG is not located in the glia limitans in either area in contrast to the ZsG signals in the glia limitans in the ventral hypothalamic area shown in panels **(A, B)** ZsG is expressed in the lateral cortical area but is located in the meninges, not the glia limitans. Scale bar on *left* applies to both panels in **(D)**.

*Dio3* expression (ZsG) was detected in the glia limitans (glial limiting membrane), which is formed by astrocyte end feet between the brain parenchyma and the meninges (**Figures 6A, B**). Functions of the glia limitans are thought to include the restriction of passage of immune cells and harmful molecules into the brain (54). *Dio3* expression (ZsG) in astrocytes of the glia limitans was indicated by co-localization with the GFAP glial marker. ZsG signals in the glia limitans were restricted to a region near the boundary between the hypothalamus and amygdala (**Figure 6C**). In the view shown, *Dio3* expression (ZsG) was also prominent in the nearby amygdaloid complex and ventral hypothalamus, as reported previously (20, 22). *Dio3* expression was not detected in the glia limitans around most of the brain parenchyma. For example, in **Figure 1**, ZsG is not expressed around all brain regions and in the regions where it is expressed, it is in the meninges. Higher magnification views in **Figure 6D** show that ZsG signals are absent in the glia limitans in representative dorsal and lateral zones around the cortical parenchyma. In the lateral zone, ZsG is detected in the meninges but not the glia limitans. The detection of *Dio3* expression in a portion of the glia limitans supports the view that *Dio3* plays a role in a range of different barrier tissues around the brain parenchyma.

### *Dio3* expression in capillary-associated cells in the immature brain

**Figure 7:**
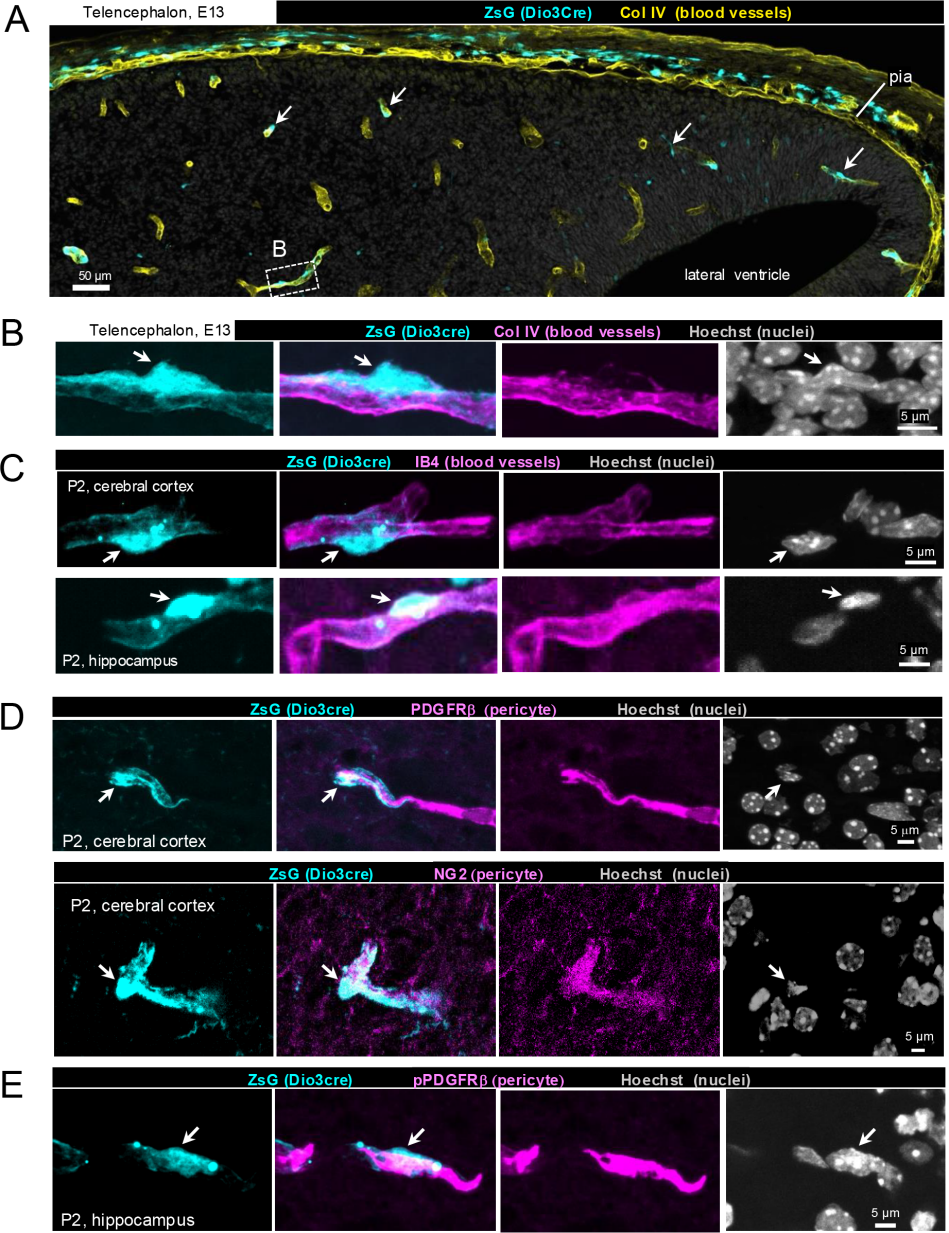
*Dio3* expression in blood vessel-associated cells of the immature brain. **(A)** *Dio3* expression (ZsG signals) in capillary-associated cells in the embryonic telencephalon at E13 (arrows); boxed region B indicates area magnified in panel **(B)** Collagen IV (Col IV) marks blood vessels in the meninges and in internal brain areas. **(B, C)** High-magnification views of examples of capillary-associated ZsG+ cells in different brain regions at E13 **(B)** and P2 **(C)**. The images show examples of ZsG+ cells in telencephalon **(B)**, cerebral cortex and hippocampal areas **(C)**. These ZsG+ cells (arrows) sit on and bulge out from the capillary and also extend irregular processes around the vessel, characteristic of pericytes. Blood vessels are indicated by Col IV or IB4 markers. Scale bars on the *right* apply to all panels in the same row. **(D, E)** ZsG+ cells (arrows) co-localized with pericyte markers PDGFRβ, NG2 **(D)** and phosphorylated-PDGFRβ **(E)**. In each row, the 4 panels show the same view imaged for different markers and for Hoechst stain (cell nuclei). Scale bars on the *right* apply to all panels in the same row.

During development, the pial vascular network around the brain extends vessels into the brain, forming an intraneural vascular plexus. These vascular networks are closely associated with pericytes which contribute to the development and maintenance of the blood-brain barrier among other functions. Pericytes display a variety of forms that extend processes around capillaries and are distributed individually along the capillary length (55). At E13, in an overview section of the telencephalon, ZsG+ cells were found associated with blood vessels in many intraneural areas (**Figure 7A**). Higher magnification indicated that these ZsG+ cells were closely attached to the adjacent blood vessel and their cell bodies bulged out from the vessel walls, which are distinctive morphological characteristics of pericytes (56) (**Figure 7B**). Examination at postnatal day 2, when blood vessels are becoming more mature, further indicated that the vessel-associated ZsG+ cells were pericytes. The nuclei of these ZsG+ cells protruded out from the capillary wall and extended processes around the vessels (**Figure 7C**). In contrast, endothelial cells that form the blood vessel wall have flatter nuclei that do not protrude from the wall and did not express detectable ZsG. Capillary-associated ZsG+ cells were observed in different brain regions including the cerebral cortex and hippocampus. **Figure 7D** and **7E** show co-staining for ZsG with pericyte markers PDGFβ, NG2 and phosphorylated PDGFRβ (pPDGFRβ), a plasma membrane marker (56). These findings suggest that *Dio3* is expressed in pericytes in the immature brain and may modulate T3 signaling at the neurovascular interface (35).

## Discussion

Our study has taken advantage of a sensitive *Dio3*^Cre^ driver-reporter system to reveal developmental and cell-specific expression of the *Dio3* gene in brain barrier tissues, including the blood-CSF and blood-brain barriers. These findings broaden the perspective on type 3 deiodinase functions in the brain to include a possible role in constraint of T3 availability at cellular interfaces between hormone-bearing fluids and immature neural tissues. The findings suggest complex regulation of T3 availability at multiple levels in the immature brain involving *Dio3* functions in barrier cell types and as previously suggested, in target neurons (11, 13, 22, 23, 57).

The functions of *Dio3* in barrier tissues are likely to involve complex cellular mechanisms and elucidating these roles will require longer term investigation. However, an interesting speculation regarding the meninges and choroid plexus is that *Dio3* regulates T3 and T4 levels while these iodothyronines are in transit through the barrier to the CSF in the ventricular and subarachnoid spaces. This is a potentially strategic step at which *Dio3* could control T4 and T3 availability in the brain. In addition to electrolytes and nutrients, CSF is thought to be a possible source of thyroid hormone for the brain (31, 47). Tracing studies have shown that in the rat brain, radio-labeled T3 or T4 introduced into the lateral ventricle is subsequently found in other ventricles, adjacent brain regions and arachnoid membranes (30). A role for *Dio3* in regulating T3 and T4 during passage through the meninges and choroid plexus implies a need for plasma membrane transporters for the uptake or release of T3 and T4 by barrier cells (28, 58). At present, it is unclear which transporters are involved because of a lack of information on these cell types at the embryonic stages when *Dio3* expression peaks. Mechanisms of transport of T3 and T4 into the CSF or brain parenchyma by different barriers are only partly understood (59) but our results suggest that transport may be coordinated with *Dio3* activity in barrier tissues.

A feature of type 3 deiodinase expression in many brain tissues is its early embryonic peak then decline by postnatal stages (16, 18, 19). The peak of *Dio3* expression in the embryonic meninges and choroid plexus at E12 - E13 (**Figure 4**), suggests a protective role for *Dio3* when immature tissues may be sensitive to damage and is consistent with early peaks of type 3 deiodinase activity in rat, mouse (17, 18) and human (19) brain regions. The subsequent decline of *Dio3* RNA expression is particularly steep in the choroid plexus before birth (**Figure 4**). Apart from regulating T4 and T3 levels while in transit through barrier tissues to the CSF, another possibility at early stages, even before barrier functions mature (32, 45), is that *Dio3* could limit T3 availability in the local environment in a form of paracrine-like control between immature cell populations. This is reminiscent of proposals that early meningeal tissue regulates retinoic acid and BMP signaling for neurogenesis in neural tissue and osteogenesis in calvarial tissue (33, 34). Deiodination has been invoked previously in paracrine-like control of T3 action in other tissues, such as testis, cochlea (16), pregnant rat uterus (24) and in the brain between glia and neurons (23). In a study of human fetal brain regions at gestational weeks 14 to 16, DIO3 immunoreactivity was described in the choroid plexus epithelium and leptomeningeal cells (60), perhaps consistent with the present results in mice.

Although *Dio3* expression declines to low or nearly-undetectable levels in some brain tissues in juvenile and adult mice, this need not exclude a subtle, continuing role for residual *Dio3* expression at these later stages. Adult-onset deletion of *Dio3* has been reported to result in subtle dysfunctions in the brain and behavior (61).

*Dio3* expression was also detected in tanycytes, which are thought to integrate T3 signaling with neuroendocrine and metabolic control (62, 63), in coordination with other factors such as retinoic acid (64) in the hypothalamus. *Dio3* expression was most prominent in α tanycytes, as indicated by Cre-mediated labeling and by analysis of spatial transcriptomics datasets (51) (**Figure 5**). Using both methods, we also investigated expression of *Dio2*, encoding a T3-amplifying deiodinase, which is involved in hypothalamic control (52, 53). *Dio2* transcripts were detected prominently in β tanycytes, consistent with previous evidence (52). Although the two deiodinase genes are expressed differentially in opposing gradients along the dorsal-ventral axis of the 3^rd^ ventricle, there is also overlapping expression, raising the possibility that both T3-inactivating (DIO3) and T3-amplifying (DIO2) enzymes act in common populations of tanycytes, although in differing proportions. The findings suggest a dynamic, dual regulation by both deiodinases in these cells. It has been proposed that tanycytes along the dorsal-ventral axis of the lining of the 3^rd^ ventricle may not exist as simple categories of α1/2 and β1/2 subtypes but may encompass a more continuous spectrum of gene expression variations (50). The differential expression of *Dio3* and *Dio2* suggest that the regulation of T3 availability may be graded similarly along the dorsal-ventral axis of the 3^rd^ ventricle rather than segregated into separate tanycyte populations.

*The Dio3* expression patterns detected suggest a wide role for type 3 deiodinase in a variety of barriers that merits future investigation. *Dio3* expression in pericytes suggests an involvement in modulating T3 availability at the blood-brain barrier as these capillary-associated cells contribute to the integrity of the barrier and microvascular stability during development (27, 35). *Dio3* was also detected in the glia limitans in a restricted region near the ventral hypothalamus (**Figure 6**). Rather than a general role in the glial limiting membrane around the brain, the results suggest a localized role, perhaps providing another layer of protection for hypothalamic areas against deleterious fluctuations of thyroid hormone levels. We note that for any of the barrier tissues where we detected *Dio3* expression, type 3 deiodinase may regulate the availability of T3 for nearby tissues but it might also regulate the differentiation or function of the barrier cells themselves as direct targets of T3 signaling.

A feature of the *Dio3* gene in many tissues is its low or moderate expression, which decreases further as development progresses (17, 19). These trends suggest that DIO3 levels must be limited because type 3 deiodinase could cause local tissue hypothyroidism if not appropriately down-regulated (8). However, low level expression presents obstacles in studying the gene. The sensitivity of *Dio3*^Cre^-mediated labeling allows detection of *Dio3* expression in barrier cell types in their natural context, which could not be achieved by previous approaches such as *in situ* hybridization (20, 21). Nonetheless, a limitation with the *Dio3*^Cre^ driver is the variable or sparse labeling of cells obtained in some tissues. Cre drivers have known inefficiencies in activating the reporter gene, which requires adequate accumulation of Cre protein in a cell over time. The steep developmental decline of *Dio3* expression could limit Cre protein from reaching a threshold for activation of the reporter. Another possibility for the particularly sparse labeling of the choroid epithelium might be that *Dio3* is expressed in a subset of the epithelial cells. This seems unlikely because although choroid plexus epithelial cells can vary with respect to developmental stage, the progenitor and differentiated cell zones are distinct (65) and do not correlate with the dispersed labeling of cells detected with the *Dio3*^Cre^ driver. Choroid plexus epithelial cells may vary due to aging or neurodegenerative conditions (66) but this is unlikely to explain sparse labeling in development. The challenges presented by low level *Dio3* expression can be partly offset by independent approaches, such as analysis of *Dio3* transcripts during development, or in some tissues, if *Dio3* expression levels are sufficient, by high resolution spatial transcriptomics. However, new or refined methods may be required for a more complete understanding of *Dio3* expression and functions in specialized cell types in development.

## Data Availability

The datasets presented in this study can be found in online repositories. The names of the repository/repositories and accession number(s) can be found below: https://www.ncbi.nlm.nih.gov/geo/, GSE315778.

## References

[1] Bernal J . Thyroid and brain: understanding the actions of thyroid hormones in brain development and function. Singapore: Bentham Science Publishers (2024).

[2] Legrand J . Effects of thyroid hormones on central nervous system development. In: Yanai J , editor.Neurobehavioral teratology. Elsevier Science Publishers, Amsterdam (1984). p. 331–63.

[3] Ng L Lyubarsky A Nikonov SS Ma M Srinivas M Kefas B . Type 3 deiodinase, a thyroid-hormone-inactivating enzyme, controls survival and maturation of cone photoreceptors. J Neurosci. (2010) 30:3347–57. doi: 10.1523/jneurosci.5267-09.2010 20203194 PMC2843520

[4] Polak M Legac I Vuillard E Guibourdenche J Castanet M Luton D . Congenital hyperthyroidism: the fetus as a patient. Horm Res. (2006) 65:235–42. doi: 10.1159/000092454 16582565

[5] Peeters RP Ng L Ma M Forrest D . The timecourse of apoptotic cell death during postnatal remodeling of the mouse cochlea and its premature onset by triiodothyronine (T3). Mol Cell Endocrinol. (2015) 407:1–8. doi: 10.1016/j.mce.2015.02.025 25737207 PMC4390549

[6] Daneman D Howard NJ . Neonatal thyrotoxicosis: intellectual impairment and craniosynostosis in later years. J Pediatr. (1980) 97:257–9. doi: 10.1016/s0022-3476(80)80487-2 7400892

[7] Bianco AC Salvatore D Gereben B Berry MJ Larsen PR . Biochemistry, cellular and molecular biology, and physiological roles of the iodothyronine selenodeiodinases. Endocr Rev. (2002) 23:38–89. doi: 10.1210/edrv.23.1.0455 11844744

[8] St Germain DL Schwartzman RA Croteau W Kanamori A Wang Z Brown DD . A thyroid hormone-regulated gene in Xenopus laevis encodes a type III iodothyronine 5-deiodinase. Proc Natl Acad Sci USA. (1994) 91:7767–71. doi: 10.1073/pnas.91.16.7767 8052658 PMC44483

[9] Peeters RP Hernandez A Ng L Ma M Sharlin DS Pandey M . Cerebellar abnormalities in mice lacking type 3 deiodinase and partial reversal of phenotype by deletion of thyroid hormone receptor alpha1. Endocrinology. (2013) 154:550–61. doi: 10.1210/en.2012-1738 23161871 PMC3529370

[10] Stohn JP Martinez ME Hernandez A . Decreased anxiety- and depression-like behaviors and hyperactivity in a type 3 deiodinase-deficient mouse showing brain thyrotoxicosis and peripheral hypothyroidism. Psychoneuroendocrinology. (2016) 74:46–56. doi: 10.1016/j.psyneuen.2016.08.021 27580013 PMC5159228

[11] Hernandez A Quignodon L Martinez ME Flamant F St Germain DL . Type 3 deiodinase deficiency causes spatial and temporal alterations in brain T3 signaling that are dissociated from serum thyroid hormone levels. Endocrinology. (2010) 151:5550–8. doi: 10.1210/en.2010-0450 20719855 PMC2954712

[12] Hernandez A Martinez ME Fiering S Galton VA St Germain D . Type 3 deiodinase is critical for the maturation and function of the thyroid axis. J Clin Invest. (2006) 116:476–84. doi: 10.1172/jci26240 16410833 PMC1326144

[13] Salas-Lucia F Fekete C Sinko R Egri P Rada K Ruska Y . Axonal T3 uptake and transport can trigger thyroid hormone signaling in the brain. eLife. (2023) 12. doi: 10.7554/elife.82683 37204837 PMC10241515

[14] Wu Z Martinez ME DeMambro V Francois M Hernandez A . Developmental thyroid hormone action on pro-opiomelanocortin-expressing cells programs hypothalamic BMPR1A depletion and brown fat activation. J Mol Cell Biol. (2023) 14. doi: 10.1093/jmcb/mjac078 36581316 PMC9982511

[15] Ng L Hernandez A He W Ren T Srinivas M Ma M . A protective role for type 3 deiodinase, a thyroid hormone-inactivating enzyme, in cochlear development and auditory function. Endocrinology. (2009) 150:1952–60. doi: 10.1210/en.2008-1419 19095741 PMC2659284

[16] Hernandez A Martinez ME Ng L Forrest D . Thyroid hormone deiodinases: dynamic switches in developmental transitions. Endocrinology. (2021) 162. doi: 10.1210/endocr/bqab091 33963379 PMC8248586

[17] Bates JM St Germain DL Galton VA . Expression profiles of the three iodothyronine deiodinases, D1, D2, and D3, in the developing rat. Endocrinology. (1999) 140:844–51. doi: 10.1210/endo.140.2.6537 9927314

[18] Kaplan MM Yaskoski KA . Maturational patterns of iodothyronine phenolic and tyrosyl ring deiodinase activities in rat cerebrum, cerebellum, and hypothalamus. J Clin Invest. (1981) 67:1208–14. doi: 10.1172/jci110136 7204575 PMC370683

[19] Kester MH Martinez de Mena R Obregon MJ Marinkovic D Howatson A Visser TJ . Iodothyronine levels in the human developing brain: major regulatory roles of iodothyronine deiodinases in different areas. J Clin Endocrinol Metab. (2004) 89:3117–28. doi: 10.1210/jc.2003-031832 15240580

[20] Escamez MJ Guadano-Ferraz A Cuadrado A Bernal J . Type 3 iodothyronine deiodinase is selectively expressed in areas related to sexual differentiation in the newborn rat brain. Endocrinology. (1999) 140:5443–6. doi: 10.1210/en.140.11.5443 10537178

[21] Martinez ME Pinz I Preda M Norton CR Gridley T Hernandez A . DIO3 protects against thyrotoxicosis-derived cranio-encephalic and cardiac congenital abnormalities. JCI Insight. (2022) 7. doi: 10.1530/ey.20.1.2 36166296 PMC9675556

[22] Liu Y Ng L Liu C Forrest D . Serotonergic and chemosensory brain areas and sensory ganglia expressing type 3 deiodinase mapped with Dio3Cre drivers. Endocrinology. (2025) 166:bqaf085. doi: 10.1210/endocr/bqaf085 40302251 PMC12092337

[23] Freitas BC Gereben B Castillo M Kallo I Zeold A Egri P . Paracrine signaling by glial cell-derived triiodothyronine activates neuronal gene expression in the rodent brain and human cells. J Clin Invest. (2010) 120:2206–17. doi: 10.1172/jci41977 20458138 PMC2877954

[24] Galton VA Martinez E Hernandez A St Germain EA Bates JM St Germain DL . Pregnant rat uterus expresses high levels of the type 3 iodothyronine deiodinase. J Clin Invest. (1999) 103:979–87. doi: 10.1172/jci6073 10194470 PMC408265

[25] Martinez ME Lary CW Karaczyn AA Griswold MD Hernandez A . Spermatogonial type 3 deiodinase regulates thyroid hormone target genes in developing testicular somatic cells. Endocrinology. (2019) 160:2929–45. doi: 10.1210/en.2019-00259 31621880 PMC6853691

[26] Rasmussen MK Mestre H Nedergaard M . Fluid transport in the brain. Physiol Rev. (2022) 102:1025–151. doi: 10.1152/physrev.00031.2020 33949874 PMC8897154

[27] Zhao Z Nelson AR Betsholtz C Zlokovic BV . Establishment and dysfunction of the blood-brain barrier. Cell. (2015) 163:1064–78. doi: 10.1016/j.cell.2015.10.067 26590417 PMC4655822

[28] Groeneweg S van Geest FS Peeters RP Heuer H Visser WE . Thyroid hormone transporters. Endocr Rev. (2020) 41. doi: 10.1210/endrev/bnz008 31754699

[29] Schweizer U Kohrle J . Function of thyroid hormone transporters in the central nervous system. Biochim Biophys Acta. (2013) 1830:3965–73. doi: 10.1016/j.bbagen.2012.07.015 22890106

[30] Dratman MB Crutchfield FL Schoenhoff MB . Transport of iodothyronines from bloodstream to brain: contributions by blood:brain and choroid plexus:cerebrospinal fluid barriers. Brain Res. (1991) 554:229–36. doi: 10.1016/0006-8993(91)90194-z 1933305

[31] Hagen GA Elliott WJ . Transport of thyroid hormones in serum and cerebrospinal fluid. J Clin Endocrinol Metab. (1973) 37:415–22. doi: 10.1210/jcem-37-3-415 4206491

[32] Saunders NR Dziegielewska KM Mollgard K Habgood MD . Physiology and molecular biology of barrier mechanisms in the fetal and neonatal brain. J Physiol. (2018) 596:5723–56. doi: 10.1113/jp275376 29774535 PMC6265560

[33] Como CN Kim S Siegenthaler J . Stuck on you: Meninges cellular crosstalk in development. Curr Opin Neurobiol. (2023) 79:102676. doi: 10.1016/j.conb.2023.102676 36773497 PMC10023464

[34] Dasgupta K Jeong J . Developmental biology of the meninges. Genesis. (2019) 57:e23288. doi: 10.1002/dvg.23288 30801905 PMC6520190

[35] Keaney J Campbell M . The dynamic blood-brain barrier. FEBS J. (2015) 282:4067–79. doi: 10.1111/febs.13412 26277326

[36] Hernandez A Martinez ME Croteau W St Germain DL . Complex organization and structure of sense and antisense transcripts expressed from the DIO3 gene imprinted locus. Genomics. (2004) 83:413–24. doi: 10.1016/j.ygeno.2003.08.024 14962667

[37] Madisen L Zwingman TA Sunkin SM Oh SW Zariwala HA Gu H . A robust and high-throughput Cre reporting and characterization system for the whole mouse brain. Nat Neurosci. (2010) 13:133–40. doi: 10.1038/nn.2467 20023653 PMC2840225

[38] Ng L Liu Y Liu H Forrest D . Cochlear fibrocyte and osteoblast lineages expressing type 2 deiodinase identified with a Dio2CreERt2 allele. Endocrinology. (2021) 162. doi: 10.1210/endocr/bqab179 34436572 PMC8475715

[39] Wang J Rattner A Nathans J . Bacterial meningitis in the early postnatal mouse studied at single-cell resolution. eLife. (2023) 12. doi: 10.7554/elife.86130 37318981 PMC10270687

[40] Ng L Liu H Liu Y Forrest D . Biphasic expression of thyroid hormone receptor TRbeta1 in mammalian retina and anterior ocular tissues. Front Endocrinol (Lausanne). (2023) 14:1174600. doi: 10.3389/fendo.2023.1174600 37033230 PMC10076699

[41] Liu Y Ng L Liu H Forrest D . Cone photoreceptor differentiation regulated by thyroid hormone transporter MCT8 in the retinal pigment epithelium. Proc Natl Acad Sci USA. (2024) 121:e2402560121. doi: 10.1073/pnas.2402560121 39018199 PMC11287251

[42] Martinez ME Charalambous M Saferali A Fiering S Naumova AK St Germain D . Genomic imprinting variations in the mouse type 3 deiodinase gene between tissues and brain regions. Mol Endocrinol. (2014) 28:1875–86. doi: 10.1210/me.2014-1210 25232934 PMC4213365

[43] DeSisto J O'Rourke R Jones HE Pawlikowski B Malek AD Bonney S . Single-cell transcriptomic analyses of the developing meninges reveal meningeal fibroblast diversity and function. Dev Cell. (2020) 54:43–59. doi: 10.1016/j.devcel.2020.06.009 32634398 PMC7769050

[44] Mancino G Sibilio A Luongo C Di Cicco E Miro C Cicatiello AG . The thyroid hormone inactivator enzyme, type 3 deiodinase, is essential for coordination of keratinocyte growth and differentiation. Thyroid. (2020) 30:1066–78. doi: 10.1089/thy.2019.0557 32111151

[45] Derk J Como CN Jones HE Joyce LR Kim S Spencer BL . Formation and function of the meningeal arachnoid barrier around the developing mouse brain. Dev Cell. (2023) 58:635–44. doi: 10.1016/j.devcel.2023.03.005 36996816 PMC10231667

[46] Lun MP Monuki ES Lehtinen MK . Development and functions of the choroid plexus-cerebrospinal fluid system. Nat Rev Neurosci. (2015) 16:445–57. doi: 10.1038/nrn3921 26174708 PMC4629451

[47] Richardson SJ Wijayagunaratne RC D'Souza DG Darras VM Van Herck SL . Transport of thyroid hormones via the choroid plexus into the brain: the roles of transthyretin and thyroid hormone transmembrane transporters. Front Neurosci. (2015) 9:66. doi: 10.3389/fnins.2015.00066 25784853 PMC4347424

[48] Dali R Estrada-Meza J Langlet F . Tanycyte, the neuron whisperer. Physiol Behav. (2023) 263:114108. doi: 10.1016/j.physbeh.2023.114108 36740135

[49] Rodriguez-Rodriguez A Lazcano I Sanchez-Jaramillo E Uribe RM Jaimes-Hoy L Joseph-Bravo P . Tanycytes and the control of thyrotropin-releasing hormone flux into portal capillaries. Front Endocrinol (Lausanne). (2019) 10:401. doi: 10.3389/fendo.2019.00401 31293518 PMC6603095

[50] Langlet F . Tanycyte gene expression dynamics in the regulation of energy homeostasis. Front Endocrinol (Lausanne). (2019) 10:286. doi: 10.3389/fendo.2019.00286 31133987 PMC6514105

[51] Yao Z van Velthoven CTJ Kunst M Zhang M McMillen D Lee C . A high-resolution transcriptomic and spatial atlas of cell types in the whole mouse brain. Nature. (2023) 624:317–32. doi: 10.1038/s41586-023-06812-z 38092916 PMC10719114

[52] Fonseca TL Correa-Medina M Campos MP Wittmann G Werneck-de-Castro JP Arrojo e Drigo R . Coordination of hypothalamic and pituitary T3 production regulates TSH expression. J Clin Invest. (2013) 123:1492–500. doi: 10.1172/jci61231 23524969 PMC3613903

[53] Fekete C Gereben B Doleschall M Harney JW Dora JM Bianco AC . Lipopolysaccharide induces type 2 iodothyronine deiodinase in the mediobasal hypothalamus: implications for the nonthyroidal illness syndrome. Endocrinology. (2004) 145:1649–55. doi: 10.1210/en.2003-1439 14684601

[54] Engelhardt B Vajkoczy P Weller RO . The movers and shapers in immune privilege of the CNS. Nat Immunol. (2017) 18:123–31. doi: 10.1038/ni.3666 28092374

[55] Attwell D Mishra A Hall CN O'Farrell FM Dalkara T . What is a pericyte? J Cereb Blood Flow Metab. (2016) 36:451–5. doi: 10.1177/0271678x15610340 26661200 PMC4759679

[56] Sweeney MD Ayyadurai S Zlokovic BV . Pericytes of the neurovascular unit: key functions and signaling pathways. Nat Neurosci. (2016) 19:771–83. doi: 10.1038/nn.4288 27227366 PMC5745011

[57] Hernandez A Stohn JP . The type 3 deiodinase: epigenetic control of brain thyroid hormone action and neurological function. Int J Mol Sci. (2018) 19. doi: 10.3390/ijms19061804 29921775 PMC6032375

[58] Mayerl S Muller J Bauer R Richert S Kassmann CM Darras VM . Transporters MCT8 and OATP1C1 maintain murine brain thyroid hormone homeostasis. J Clin Invest. (2014) 124:1987–99. doi: 10.1172/jci70324 24691440 PMC4001533

[59] Alevyzaki A Markova B De Angelis M Muller TD Chandrasekar A Muller-Fielitz H . Inactivation of thyroid hormone transporters Mct8/Oatp1c1 in mouse brain endothelial cells causes region-specific alterations in central thyroid hormone signaling. Thyroid. (2025) 35:816–27. doi: 10.1089/thy.2025.0089 40622283

[60] Lopez-Espindola D Garcia-Aldea A Gomez de la Riva I Rodriguez-Garcia AM Salvatore D Visser TJ . Thyroid hormone availability in the human fetal brain: novel entry pathways and role of radial glia. Brain Struct Funct. (2019) 224:2103–19. doi: 10.1007/s00429-019-01896-8 31165302

[61] Stohn JP Martinez ME St Germain DL Hernandez A . Adult onset of type 3 deiodinase deficiency in mice alters brain gene expression and increases locomotor activity. Psychoneuroendocrinology. (2019) 110:104439. doi: 10.1016/j.psyneuen.2019.104439 31561084 PMC7259167

[62] Mullur R Liu YY Brent GA . Thyroid hormone regulation of metabolism. Physiol Rev. (2014) 94:355–82. doi: 10.1152/physrev.00030.2013 24692351 PMC4044302

[63] Zhang Z Boelen A Bisschop PH Kalsbeek A Fliers E . Hypothalamic effects of thyroid hormone. Mol Cell Endocrinol. (2017) 458:143–8. doi: 10.1016/j.mce.2017.01.018 28088468

[64] Stoney PN Helfer G Rodrigues D Morgan PJ McCaffery P . Thyroid hormone activation of retinoic acid synthesis in hypothalamic tanycytes. Glia. (2016) 64:425–39. doi: 10.1002/glia.22938 26527258 PMC4949630

[65] Dani N Herbst RH McCabe C Green GS Kaiser K Head JP . A cellular and spatial map of the choroid plexus across brain ventricles and ages. Cell. (2021) 184:3056–74. doi: 10.1016/j.cell.2021.04.003 33932339 PMC8214809

[66] Murakami R Ueno M . Morphological and molecular characteristics of choroid plexus epithelium in aged brains. Int J Mol Sci. (2026) 27. doi: 10.3390/ijms27052505 41828720 PMC12986088

